# Supplementary material for: Reframing the contribution of pelagic Sargassum epiphytic N2 fixation
Source: PLoS One. 2023 Aug 1;18(8):e0289485. doi: 10.1371/journal.pone.0289485 (PMC10393174; doi:10.1371/journal.pone.0289485)
Supplement: Attachment — Submitted filename: Response to Reviewers.docx [file pone.0289485.s002.docx]

**Response to Reviewers**

Dr. García,

Thank you so much for lending your time and expertise to the review of our manuscript and for your persistence in finding a reviewer for our paper—we realize it is not always an easy or straightforward task. We greatly appreciate your service to this journal and your continued efforts toward the publication of our manuscript.

Thank you,

Claire (on behalf of all authors)

**Journal requirements:**

1. Please ensure that your manuscript meets PLOS ONE's style requirements, including those for file naming.

**Response:** I have changed the figure titles and their in-text citations from “Figure” to “Fig” as required.

2. We note that Figure 1 in your submission contain [map/satellite] images which may be copyrighted. All PLOS content is published under the Creative Commons Attribution License (CC BY 4.0), which means that the manuscript, images, and Supporting Information files will be freely available online, and any third party is permitted to access, download, copy, distribute, and use these materials in any way, even commercially, with proper attribution. For these reasons, we cannot publish previously copyrighted maps or satellite images created using proprietary data, such as Google software (Google Maps, Street View, and Earth). For more information, see our copyright guidelines: <http://journals.plos.org/plosone/s/licenses-and-copyright>.

**Response:** I have changed the basemap from the ArcMap Light Gray Canvas to USGS’s publicly available “Small-scale Dataset-1:1,000,000-Scale State Boundaries of the United States 201403 Shapefile (available from: <https://www.sciencebase.gov/catalog/item/581d052de4b08da350d524e5>).

### I have also included a citation for the bathymetry contour used in this figure. It was generated from GEBCO’s publicly available 2023 gridded bathymetry data (doi: 10.5285/f98b053b-0cbc-6c23-e053-6c86abc0af7b). I have updated my reference list to reflect this change.

3. Please review your reference list to ensure that it is complete and correct. If you have cited papers that have been retracted, please include the rationale for doing so in the manuscript text, or remove these references and replace them with relevant current references. Any changes to the reference list should be mentioned in the rebuttal letter that accompanies your revised manuscript. If you need to cite a retracted article, indicate the article’s retracted status in the References list and also include a citation and full reference for the retraction notice.

**Response:** Reference list is complete and correct. I have also added DOIs to all references where they are available.

**Reviewers’ comments:**

4. Line 127. In the table, please clarify what n means. I am assuming that it means that either 2 or 3 acetylene reduction samples were run, but clarify in the Table heading.

**Response:** Yes, n stands for the number of replicate assays. This explanation has been added to Table 1’s title.

5. Line 124. Figure legend 1, what is the black squiggly line? Maybe the edge of the Continental Shelf? If so, state the depth represented by the line. 200 meters?

**Response:** The black like represents the 250-meter isobath. Figure 1’s legend has been updated to include this as well as a citation for the data used to generate it.

6. Line 134 & 148. Can you state the u mol photons per m2/ sec of PAR that the samples were exposed to? This would make it easier for someone to duplicate your research rather than trying to purchase an identical set of light bulbs.

**Response:** I have replaced the light bulb’s brand name with µmol photons m^-2^ s^-1^ PAR in both places.
